# Socio-demographic factors of cesarean births in Nha Trang city, Vietnam: a community-based survey

**DOI:** 10.1186/s41182-020-00239-2

**Published:** 2020-07-10

**Authors:** Mizuki Takegata, Carine Ronsmans, Hien Anh T. Nguyen, Noriko Kitamura, Chihiro Iwasaki, Michiko Toizumi, Hiroyuki Moriuchi, Duc Anh Dang, Lay-Myint Yoshida

**Affiliations:** 1 Department of Pediatric Infectious Diseases, Institute of Tropical Medicine, Nagasaki University, 1-12-4 Sakamoto, Nagasaki, 852-8523 Japan; 2 Department of Infectious Disease Epidemiology, London School of Hygiene and Tropical Medicine, London, WC1E 7HT UK; 3 National Institute of Hygiene and Epidemiology, Hanoi, 100000 Vietnam; 4 Department of Pediatrics, Nagasaki University Hospital, Nagasaki, 852-8523 Japan

**Keywords:** Cesarean birth rate, Community-based mothers, Socio-demographic factors, Urban city, Vietnam

## Abstract

**Background:**

The cesarean section rate in Vietnam has been increasing especially in urban area. However, limited evidence identified regarding socio-demographic factors of the cesarean section birth. The objective of this study was to determine the current cesarean birth rate and the associated socio-demographic factors among mothers in Nha Trang city, south-central Vietnam.

**Methods:**

A community-based cross-sectional study was conducted between October and November in 2016 as part of a *Streptococcus pneumoniae* carriage survey conducted in 27 communes of Nha Trang city. From each commune, 120 mothers and their children less than 2 years old were randomly selected. Mothers were asked to answer standardized questions regarding socio-demographic information and mode of birth. Multivariate logistic regression was adopted to examine associations between socio-demographic variables and mode of birth.

**Results:**

Of 3148 participants, the number of cesarean births was 1396 (44.3 %). Older maternal age (≥ 30 years old), having another child going to school or kindergarten, monthly income more than 644 USD, gestational weeks at birth over 42 weeks, and low (< 2500 g) or high (≥ 3500 g) birth weight were associated with higher likelihood of cesarean births.

**Conclusion:**

The CS rate obtained in this study was more than twice of what is recommended by the World Health Organization, which is consistent with the previous nation-wide study in Viet Nam. Further monitoring is suggested to examine the non-medical reason for the increased CS rate.

## Background

Rates of cesarean section (CS) have increased dramatically over the last 30 years, especially in middle- and high-income countries [1–3]. Although deciding on what should be considered an acceptable CS rate has been controversial, there is no evidence that CS rates higher than 10% decrease maternal or neonatal mortality [4]. Nevertheless, the World Health Organization (WHO) has recommended an optimal CS rate between 10% and 15% [5]. Although there is little evidence to support this rate recommended by the WHO, a systematic review by Betran et al. [6] revealed that rates of CS higher than the threshold do not reduce mortality. However, many countries have CS rates much higher than this recommended threshold [1, 2]. Several studies have suggested that unnecessary CS could increase maternal and neonatal morbidity [4, 7], including postpartum complications [7], pregnancy complications [7], and neonatal risks [7, 8]. A recent meta-analysis has shown that children born by CS had an increased risk of asthma up to the age of 12 years and obesity up to the age of 5 years [8].

Betran et al. [2] compared the CS rates among 150 countries and reported that the highest CS rates were found in Latin America and the Caribbean region (40.5%), followed by North America (32.3%), Oceania (31.1 %), Europe (25%), Asia (19%), and Africa (7.3%). CS rates in Asia are rapidly increasing with an annual rise of between 4.4% and 8.5% [2]. In Asia, Vietnam has the fifth-highest CS rate (2014, 28%) after China (2013, 41%), Republic of Korea (2007, 37%), Thailand (2015, 33%), and Sri Lanka (2014, 32%) according to a multiple indicator cluster survey (MICS) [9]. A rapid increase in CS rates has also been observed in Vietnam from 10% in 2002 to 28% in 2014 [9]. This increase in CS rates has been especially high in urban areas (2014, 43%).

Previous studies have investigated the determinants of increased CS rates in several Asian countries, such as China [10], Bangladesh [11], India [12], Nepal [13], and Cambodia [14]. The determinants of CS have included various dimensions related to the physical, social, cultural, and psychological characteristics of women and their families as well as factors relating to the health system in the study area. Physical characteristics included older maternal age [1, 10, 13] and primiparity [10]. Socio-demographic factors included higher education and higher economic status of mothers and their families [1, 10, 13]. The parental or maternal request for CS without a medical indication has been reported in China [15], Cambodia [14], and Nepal [13]. In addition, pregnancies with a male fetus, access to full-insurance payments, and pregnancies after infertility treatment were associated with CS in China [15].

A few studies have been conducted that have examined the determinants of CS in Vietnam. For example, Loezenien et al. [16] conducted a secondary analysis using country-wide data of 1350 participants in a 2014 MICS survey. The authors claimed that there was a large gap in CS rates between urban (42.4%) and rural areas (22.9%) and that factors associated with CS rates included maternal age at delivery over 35 years and higher economic background [16].

Most previous studies have focused on the northern part of Vietnam. Two population studies [17, 18] conducted in Quang Ninh province close to the border of China found that mothers of a male child were more likely to receive CS surgery than mothers giving birth to a female child [17]. This finding likely stems from the enactment of the one-or-two child policy in Vietnam in the late 1980s [17, 19]. Another two population studies [20, 21] conducted in urban and suburban areas of Hanoi also reported a difference in the CS rate between male and female children. Hence, these authors concluded that high income and differences in the CS rates between the sexes might be related to requests made for CS without medical needs [16, 17].

Fewer studies have been conducted in the central or southern regions of Vietnam compared with the northern region. Giang et al. [22] investigated CS rates and their determinants in 10 hospitals in Da Nang city, an urban area in central Vietnam. The overall CS rate was 58.6%, and private hospitals had a much higher rate of CS (70.6%). CS rates were higher in women that were older than 30 years, had a history of abortion, worked an office job, were pregnant with a male child, or gave birth to a baby of heavier birth weight.

Given that a preference for CS for male children might be influenced in the northern part of Vietnam by the culture of “Confucianism,” which originated in China, compared with the southern part of the country, geographical differences in several factors might contribute to variation in CS rates. In addition, more data in urban settings are needed to assess the consistency of a previous analysis of MICS data and confirm the pattern that CS rates are higher in urban settings compared with rural ones [9].

The objective of this study was to identify the socio-demographic factors and delivery characteristics associated with CS among mothers living in Nha Trang city, Vietnam.

## Methods

This study was conducted in Nha Trang city, the capital of Khánh Hòa province, which is located 445 km north of Ho Chi Minh City in Vietnam. The estimated population in 2020 was 329,373 [23]. Nha Trang city has become one of the main holiday destinations in Vietnam and has been experiencing economic growth. There is one public tertiary hospital and three private hospitals where CS can be performed.

This study was completed as a part of a *Streptococcus pneumoniae* carriage survey conducted in Nha Trang city across 27 communes: 8 (30%) suburban and 19 (70%) urban communes. We conducted a cross-sectional survey in the 27 communes in October and November 2016. First, each community health center made a list of all children aged 4 to 11 months and 14 to 23 months and their mothers living in the commune. All newborn babies must register at CHC for free national health insurance to receive national immunization program vaccines. We used this data since these newborn children data at CHC is the most representative data for number of deliveries in Nha Trang city. Because this survey originally aimed to investigate the carriage rate of *Streptococcus pneumococcus* among participants, we excluded children that were born with a cleft lip or cleft palate and their mothers due to the difficulty of collecting nasopharyngeal samples. Second, children and their mothers were randomly selected from this list and invited to participate in the pneumococcal carriage survey until 120 participants per commune had been recruited. Because of the ubiquity of cell phone use in urban areas of Vietnam, trained staff from community health centers made a phone call to the candidates and invited them to participate. Home visit was made and requested to participate in the study for those who do not have mobile phone. One or two staff members visited interested mothers, explained the study, and obtained written informed consent. Upon agreement, the mother was then interviewed using a structured questionnaire requesting socio-demographic information.

In terms of sample size calculation, we assumed the true proportion of children aged < 24 months who have *S. pneumoniae* in the nasopharynx in the study area to be 0.5, to obtain the largest sample size, and required the estimate to be within 0.04 of the true value (precision) within the 95% confidence interval (CI). Based on this, the minimum sample size was calculated to be 600 for each introduction arm (6 communes). As there were six communes (clusters) in each arm, we decided to recruit 120 per communes resulting in 720 per arm, and 3240 for 27 communes.

Appendix Fig. 1 shows a flow chart of participants. Out of 5533 pairs listed in 27 commune health centers, 5348 were invited to the study by means of a phone call from community health center staff. Around one out of five candidates (*n* = 1219, 22.8%) refused to participate in this study, and 17% (*n* = 952) could not be contacted. In addition, 29 pairs were not eligible (duplicate participants or out of the eligible age range) based on the participant criteria. A total of 3148 pairs of mothers and their children participated in the survey: 1562 children were aged 4 to 11 months, and 1586 were aged 14 to 23 months.

The questionnaire included socio-demographic variables, such as maternal age, educational background of the mother and the head of household, name of the commune of residence, household income in the previous month (Vietnamese don, VND), whether the mother has another child going to school or kindergarten, and the total number of household members. Household income in the previous month (VND) was converted into US dollars (1 dollar = 23,291 VND, Sep. 17th, 2019). Living in suburban or urban areas was coded based on the administrative definition of the communes. Delivery information included the mode of delivery, the child’s sex and birth weight (g), gestational weeks at birth, and the date of birth. The day of delivery was calculated from the birth date of the child. Matshidze et al. [24] observed that the CS rate was much higher during weekdays than during the weekends in private settings, whereas CS rates in public facilities did not vary throughout the week, suggesting that there is an institutional effect related to the scheduling of planned CS at convenient times for obstetricians.

Binary logistic regression was performed to examine associations between socio-demographic variables and the mode of delivery. The adjusted model included variables that were independently associated with CS births in the crude analysis (*p* < 0.05). To assess the multicollinearity of independent variables, variables that showed moderate correlations with others (*r* ≥ 0.4) were excluded from the final model [25]. Analyses were conducted in STATA version 14.0 (Stata Corp., College Station, TX, USA).

This study was approved by the ethical review boards of the Institute of Tropical Medicine, Nagasaki University, Japan, and the National Institute of Hygiene and Epidemiology, Hanoi, Vietnam.

## Results

A total of 1396 out of 3148 births were completed by CS, resulting in a CS rate of 44.3% (Table 1). Of the 1752 mothers that gave birth vaginally, 227 (12.9%) reported that they had received a medical intervention, such as forceps, vacuum, or oxytocin induction. Of the 1396 CS births, 603 (43.1%) were planned during pregnancy and 777 (55.6%) were decided during labor. Previous CS was the most common indication (*n* = 420, 30.1%) followed by cephalo-pelvic disproportion (CPD) (*n* = 157, 11.2 %), oligohydramnios (*n* = 147, 10.5%), and malposition (*n* = 128, 9.1%).

**Table 1:** Mode of birth (*n* = 3148)

	*N*	(%)
**Vaginal delivery**	**1752**	**(55.6)**
Spontaneous vaginal delivery	1314	(75.0)
Induced vaginal delivery	227	(12.9)
Unknown	211	(12.1)
Induced vaginal delivery		
Drug-induced vaginal delivery	198	(11.3)
Instrumental vaginal delivery	4	(0.2)
Unknown	25	(1.4)
**Cesarean section**	**1396**	**(44.3)**
Medically planned cesarean section	603	(43.2)
Emergency cesarean section	777	(55.7)
Unknown	16	(1.1)
Reasons for cesarean section		
Previous cesarean section	420	(30.0)
Cephalopelvic disproportion	157	(11.2)
Oligohydramnios	147	(10.5)
Malposition	128	(9.1)
Fetal distress	103	(7.3)
Ineffective contraction	89	(6.3)
Post-term delivery	62	(4.4)
Premature rupture of the membrane	48	(3.4)
Hypertension during pregnancy	43	(3.0)
Anomaly of placenta	39	(2.7)
Coiling or short umbilical cord	10	(0.7)
Twins	3	(0.2)
Other	56	(4.0)
Unknown	91	(6.5)

Table 1 shows obstetric information of last previous delivery in the participants. Obstetric information included mode of delivery and reasons for cesarean section. Number (percentage) in each variable is displayed in the table

Table 2 shows the characteristics of the participants and the CS birth rates in each group. Few mothers were younger than 20 years old (*n* = 70, 2.2%), and 517 (16.4%) were older than 35. A small number of mothers (*n* = 167, 5.3%) had not completed primary school, while 1207 (38.3 %) had received post-secondary education. Approximately one-fifth of mothers (*n* = 707, 22.4 %) reported a monthly household income in the previous month higher than 644 USD. Few mothers (*n* = 85, 2.7%) gave birth post-term (≥ 42 gestational weeks). Approximately one-third of babies (*n* = 1116, 35.4 %) were born weighing over 3500 g at birth, while the number of babies weighing less than 2500 g at birth was 69 (2.1%). The percentages of CS births were similar among each day of the week (ranging from 13.1 to 15.2%). The number of male babies (*n* = 1660, 53.7%) was slightly higher than the number of female babies (*n* = 1448, 47.2%).

**Table 2:** Sociodemographic determinants of CS birth (*n* = 3148)

	Number of births *N* (%)		Cesarean section rate (%)	Model 1		Model 2	
				Crude OR^1^	95% CI^2^ for OR	Adjusted OR^1^	95% CI^2^ for OR^1^
**Demographic information of mothers**							
Age group of mother							
< 20 years of age	70	(2.2)	22.9	0.57	(0.34, 1.03)	0.6	(0.33, 1.11)
20-24 years of age	508	(16.1)	34.1	1		1	
25-29 years of age	1095	(34.7)	39.8	**1.28**	(**1.46**, **2.29**)	1.19	(0.94, 1.49)
30-34 years of age	958	(30.4)	48.6	**1.83**	(**1.46**, **2.29**)	**1.82**	(**1.43**, **2.31**)
≥ 35 years of age	517	(16.4)	59.0	**2.78**	(**2.16**, **3.60**)	**2.9**	(**2.22**, **3.79**)
Educational background of mother							
Unfinished primary	167	(5.3)	40.1	1.04	(0.71,1.50)	1.01	(0.68, 1.21)
Primary	396	(12.5)	39.1	1		1	
Lower secondary	584	(18.5)	39.2	1.00	(0.77, 1.30)	1.03	(0.84, 1.38)
Upper secondary	794	(25.2)	43.3	1.88	(0.92, 1.51)	1.16	(0.90, 1.51)
Post-secondary or higher	1207	(38.3)	49.8	**1.54**	(**1.22**, **1.94**)	1.35	(0.81, 1.37)
Mother who does not have another child going to kindergarten or school	1238	(39.3)		1		1	
Mother who has another child going to kindergarten or school	1910	(60.6)	42.8	**0.85**	(**0.74**, **0.98**)	**0.73**	(**0.62**, **0.86**)
**Family and residential information**							
Residential area							
Suburban area	958	(30.4)	40.3	1		1	
Urban area	2190	(69.5)	46.1	**1.26**	(**1.08**, **1.47**)	0.89	(0.76, 1.05)
Monthly income^3^							
< 342 USD (< 8 million VND)	974	(30.9)	41.2	1		1	
343-428USD (8-9 million VND)	820	(26.0)	44.4	1.14	(0.94, 1.37)	1.08	(0.88, 1.31)
429-643USD (10-14 million VND)	647	(20.5)	42.8	1.06	(0.87, 1.30)	1.09	(0.87, 1.35)
≥ 644 USD (≥ 15 million VND)	707	(22.4)	50.1	**1.43**	(**1.17**, **1.74**)	**1.43**	(**1.14**, **1.80**)
Educational background of head of household							
Unfinished primary	477	(15.1)	37.7	0.92	(0.72, 1.17)		
Primary	671	(21.3)	39.6	1			
Lower secondary	529	(16.8)	43.9	1.18	(0.94, 1.49)		
Upper secondary	698	(22.1)	46.6	**1.32**	(**1.07**, **1.64**)		
Post-secondary or higher	773	(24.5)	50.8	**1.57**	(**1.27**, **1.94**)		
Household size (no. of household members)							
< 5	1122	(35.6)	48.5	1		1	
5-9	1851	(58.8)	41.9	**0.76**	(**0.66**, **0.89**)	0.82	(0.71, 0.97)
≥ 10	175	(5.5)	43.4	0.81	(0.59, 1.94)	0.85	(0.60, 1.23)
**Birth outcome**							
Gestational weeks							
< 37 weeks 0 day	173	(5.5)	45.7	1.08	(0.79, 1.47)	1.03	(0.73, 1.46)
37 weeks 0 day-41w 6 days	2890	(91.8)	43.6	1		1	
≥ 42 weeks 0 day	85	(2.7)	65.9	**2.49**	(**1.58**, **3.92**)	**2.62**	(**1.63**, **4.21**)
Birth weight of child							
< 2500 g	69	(2.1)	55.1	**1.99**	(**1.19**, **3.32**)	**1.87**	(**1.09**, **3.26**)
2500-2999 g	457	(14.5)	38.1	1		1	
3000-3499 g	1506	(47.8)	40.	1.08	(0.87, 1.34)	1	(0.79, 1.25)
3500-3999 g	910	(28.9)	49.8	**1.61**	(**1.28**, **2.02**)	**1.5**	(**1.18**, **1.91**)
4000-4499 g	180	(5.7)	59.4	**2.38**	(**1.67**, **3.39**)	**2.04**	(**1.42**, **2.95**)
≥ 4500 g	26	(0.8)	80.8	**6.83**	(**2.52**, **18.44**)	**5.57**	(**2.02**, **15.34**)
Day of the week at birth							
Monday	476	(15.1)	48.3	1			
Tuesday	480	(15.2)	45.0	0.87	(0.67, 1.12)		
Wednesday	442	(14.0)	46.2	0.91	(0.70, 1.18)		
Thursday	451	(14.3)	46.3	0.92	(0.71, 1.19)		
Friday	461	(14.6)	42.7	0.79	(0.61, 1.03)		
Saturday	413	(13.1)	41.9	0.77	(0.59, 1.00)		
Sunday	425	(13.5)	39.3	0.69	(0.53, 0.90)		
Child’s sex							
Male	1660	(52.7)	44.5	1			
Female	1448	(47.2)	45.4	0.99	(0.86, 1.14)		

Table 2 shows number of birth (percentage) and cesarean section rate in socio-demographic, obstetric, and institutional variables. Model 1 displays the results of crude odds ratio (Crude OR) and confidential interval (CI) of the socio-demographic, obstetric, and institutional variables for cesarean section rate. Model 2 displays the results of adjusted OR (AOR) and CI of significant variables in model 1 for cesarean section rate

^1^*OR* odds ratio, bold number, statistical significant

^2^Confidence interval

^3^1USD = 23,291 VND (17th Sep. 2019), each category was divided by four quantiles (25, 50, and 25 percentiles)

Rates of CS were high among women older than 35 years (59.0%), those that had completed post-secondary education (49.8%), those that had a monthly income greater than 644 USD (50.1%), and those with babies weighing over 3500 g (3500–3999 g, 49.8%; 4000–4499 g, 59.4%; > 4500 g, 80.8%) or under 2500 g (55.1%). The CS rate varied depending on the day of the week (ranging from 39.3 to 48.3%), but the sex of the child had no effect on the CS rate.

A moderate correlation was observed between the educational backgrounds of the mother and the head of household (*r* = 0.57), which was assumed to be a multicollinear factor. After adjustment for potential confounding factors, CS rates were higher among older women (30–34 years, adjusted odds ratio [AOR] = 1.18, 95% confidence interval [CI] (1.43, 2.31); ≥ 35 years, AOR 2.90, 95% CI (2.22, 3.79)); those with a monthly income of 644 USD or more (AOR = 1.43, 95% CI = 1.14, 1.80), those giving birth after 42 weeks of gestation (AOR = 2.62, 95% CI = 1.63, 4.21), those with babies weighing less than 2500 g at birth (AOR = 1.87, 95% CI = 1.09, 3.26), and those with babies weighing 3500 g or more at birth (3500–3999 g, AOR = 1.50, 95% CI = 1.18, 1.91; 4000–4499 g, AOR = 2.04, 95% CI = 1.42, 2.95; ≥ 4500 g, AOR = 5.57, 95% CI = 2.02, 15.34).

## Discussion

The CS rate in Nha Trang city in Vietnam was 44.3%. The rate of CS was particularly high among women over the age of 30 years, those with a monthly income of more than 644 USD, those giving birth after 42 weeks of gestation, and those with either a low (< 2500 g) or high (≥ 3500 g) birth weight baby.

The CS rate (44.3%) observed in our study is consistent with the rate observed in a national survey of urban areas in Vietnam in 2014 [9]. In addition, the rate is much higher than the CS rate recommended by the WHO (10–15%) [5], suggesting that there may be a substantial number of unnecessary CS births that are not based on medical need. The CS rate in urban areas of Vietnam has increased dramatically over the last 20 years from 12% in 1997 (Demographic Health Survey [DHS] 1997) to 23%, 31%, and 43% in 2002, 2010, and 2014, respectively (DHS 2002, MICS 2010–2011, MICS 2014) [9]. Giang et al. [22] noted that the CS rate at private hospitals (70.6%) was much higher than that at public hospitals (57.9%). After the economic reform in the late 1980s, private health facilities have increased in number dramatically in metropolitan areas, attracting wealthier people that expect better quality care in private hospitals. The link between the mode of delivery at private hospitals and the risk for CS has been widely documented in other countries [1]. One of the limitations of our study is that we did not ask mothers whether they gave birth at private or public hospitals; nevertheless, our sample covered three private hospitals. Thus, the high rate of CS may have reflected the increased use of CS associated with private hospitals.

Mothers with higher economic status were more likely to receive CS births. The health care system was organized by the Vietnamese government in the late 1980s [26]. Currently, health insurance covers a variety of health care services, including from 80 to 100% of the total cost of antenatal checkups and both normal and abnormal childbirths. The introduction of user fees for service in the late 1980s has increased the number of service options [26] as well as the out-of-pocket expenses of patients. Mothers that undergo CS sometimes need to pay out-of-pocket expenses for additional services, such as a single room charge for hospitalization; therefore, mothers with a low economic status may not be able to afford medical expenses for CS [27]. Another reason for the association between higher economic status and CS birth may be a difference in access to antenatal care services between women with higher and lower socio-economic status. Wealthier pregnant women receive more frequent and qualified antenatal care, including ultrasonographic examinations that can aid the identification of medical conditions requiring CS [27].

Contrary to a previous study reporting higher CS rates in urban areas (43%) than in suburban areas (21%) [9], place of residence was not significantly associated with CS rates in our final adjusted model. This discrepancy may stem from the greater proximity between urban and suburban regions inside Nha Trang city. As several studies have indicated [1, 13], one of the reasons for higher CS rates in urban areas is increased accessibility to maternity health care facilities that are well equipped and well-staffed. Another reason is that patients with higher socio-economic status who are able to receive CS often live in urban areas [3, 27]. However, only a weak association of education and economic status with residential areas was observed in our study. Thus, the higher geographical proximity among the study areas likely explained the negligible difference in the CS rate between urban and suburban areas.

Child sex was not associated with the CS rate in our study in contrast to findings from previous studies in Vietnam [17, 22]. Giang et al. [22] suggested that some families under the traditional culture of “Confucianism” may request a CS when they find out by ultrasonography that the child is a male. The sex ratio at birth was higher in northern areas (> 120 male births to 100 female births) than in central or southern areas of Vietnam (< 110 male births to 100 female births) [28]. Da Nang city is located approximately 500 km north of Nha Trang city. Therefore, the association between child sex and CS may be influenced by cultural differences between regions.

Older maternal age (≥ 30 years old), low birth weight (< 2500 g), and high birth weight (≥ 3500 g) resulted in higher CS rates. Although we did not take data on birth parity in our study, CS rates were lower for mothers that reported “having another child going to school or kindergarten.” These findings are consistent with previous studies that have been conducted in other countries [29, 30]. Older age, primiparity, low birth weight, and high birth weight may be associated with a higher risk of pregnancy and birth complications and a greater need for CS [27]. However, given that the CS rates in younger age groups (20–29 years old) were over 30%, CS for a substantial number of patients might not have been medically required.

The major strength of our study was that we recruited a large number of participants (*n* = 3140) and followed a strict randomization enrollment technique. Our data included participants from urban and suburban areas across Nha Trang city.

Our study has a few limitations that should be considered. First, 22.8% of participants refused to participate in the *Streptococcus pneumococcus* carriage survey. The lack of participation by some participants might have reflected their fear of having a nasopharyngeal swab taken from their child regardless of their social-economic status. In addition, we were unable to contact 17.9% of the candidates on the original candidate list, possibly because they had changed their mobile phones or moved to a different address. These facts might slightly influence the representativeness of participants. Second, we did not have information on parity and other clinical factors, such as pregnancy, delivery complications, and the number of antenatal checkups identified in a previous study [16], as this study was a part of a *Streptococcus pneumoniae* carriage survey. Third, our data were based on the ability of mothers to recall the requested information, but we were unable to confirm the information that they provided from medical charts. Although the medical reasons for CS in our study were similar to those provided by a previous study [22], data based on clinical indications for CS from hospital records would have been superior and of higher quality than the data that we collected. Women may have also misreported their age, income, gestational age, or the birth weight of their children. Misclassification of gestational age and birth weight may have been related to the mode of delivery, although it is difficult to predict in which direction this would have biased the results.

## Conclusions

The CS birth rate in Nha Trang city was 44.3% in 2016. Older maternal age (≥ 30 years old), having another child, monthly income greater than 644 USD, gestational weeks at birth over 42 weeks, and low (< 2500 g) and high (≥ 3500 g) birth weight was associated with a higher likelihood of CS birth.

## Figures and Tables

**Fig. 1:**
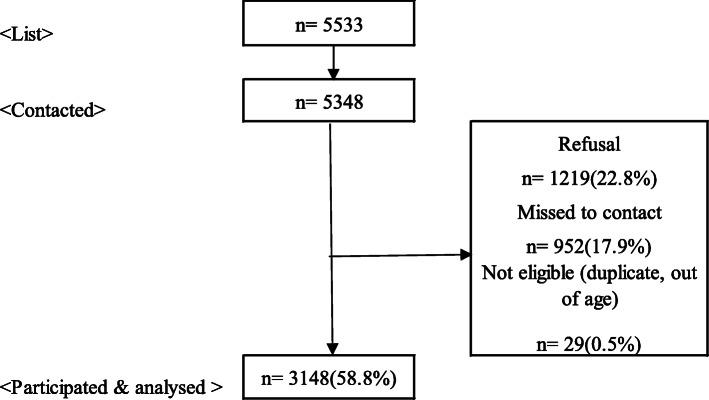
Flow chart of participants. The figure displays the number of candidate in the list of 27 commune health centers, the number of contacted candidates, and the number of final participants. The number of refusal, that of missed to contact, and that of non-eligible were regarded as drop out

## Data Availability

The database used for this study is part of an ongoing PCV reduced dosing schedule trial in Nha Trang, Vietnam. The data can be assessed by contacting Prof. Lay Myint Yoshida (lmyoshi@nagasaki-u.ac.jp) who is the PI of the project.

## Abbreviations

AOR: Adjusted odds ratio
CI: Confidential interval
CPD: Cephalo pelvic disproportion
CS: Cesarean section
DHS: Demographic health surveys
MICS: Multiple indicator cluster surveys
*N*: Number
WHO: World Health Organization
USD: US dollar
VND: Vietnamese don

## References

[1] Leone T, Padmadas SS, Matthews Z (2008). Community factors affecting rising caesarean section rates in developing countries: an analysis of six countries. Soc Sci Med..

[2] Betran AP, Ye J, Moller AB, Zhang J, Gulmezoglu AM, Torloni MR (2016). The increasing trend in caesarean section rates: global, regional and national estimates: 1990-2014. PLoS One..

[3] Ronsmans C, Holtz S, Stanton C (2006). Socioeconomic differentials in caesarean rates in developing countries: a retrospective analysis. Lancet..

[4] Ye J, Zhang J, Mikolajczyk R, Torloni MR, Gülmezoglu AM, Betran AP (2016). Association between rates of caesarean section and maternal and neonatal mortality in the 21st century: a worldwide population-based ecological study with longitudinal data. BJOG..

[5] WHO statement on caesarean section rates. Reprod Health Matters. 2015;23(45):149-50. doi: 10.1016/j.rhm.2015.07.007. 10.1016/j.rhm.2015.07.007 26278843

[6] Betran AP, Torloni MR, Zhang J, Ye J, Mikolajczyk R, Deneux-Tharaux C (2015). What is the optimal rate of caesarean section at population level? A systematic review of ecologic studies. Reprod Health..

[7] Sandall J, Tribe RM, Avery L, Mola G, Visser GH, Homer CS (2018). Short-term and long-term effects of caesarean section on the health of women and children. Lancet..

[8] Keag OE, Norman JE, Stock SJ (2018). Long-term risks and benefits associated with cesarean delivery for mother, baby, and subsequent pregnancies: systematic review and meta-analysis. PLoS Med..

[9] United Nations Children’s Fund. In: UNICEF Data: monitoring the situation of children and women 1954. https://data.unicef.org/topic/maternal-health/delivery-care/ Accessed 1 April 2018.

[10] Lei H, Wen SW, Walker M (2003). Determinants of caesarean delivery among women hospitalized for childbirth in a remote population in China. JOGC.

[11] Sreevidya S, Sathiyasekaran BW (2003). High caesarean rates in Madras (India): a population-based cross sectional study. BJOG..

[12] Anwar I, Nababan HY, Mostari S, Rahman A, Khan JAM. Trends and inequities in use of maternal health care services in Bangladesh, 1991-2011. PLoS ONE. 2015;10(3). 10.1371/journal.pone.0120309. 10.1371/journal.pone.0120309 PMC4370698 25799500

[13] Prakash KC, Neupane S (2014). Cesarean deliveries among Nepalese mothers: changes over time 2001-2011 and determinants. Arch Gynecol Obstet..

[14] Schantz C, Sim KL, Petit V, Rany H, Goyet S (2016). Factors associated with caesarean sections in Phnom Penh. Cambodia. Reproductive Health Matters..

[15] Feng XL, Wang Y, An L, Ronsmans C (2014). Cesarean section in the People’s Republic of China: current perspectives. International Journal of Women's Health..

[16] Loenzien M, Schantz C, Luu BN, Dumont A (2019). Magnitude and correlates of caesarean section in urban and rural areas: a multivariate study in Vietnam. PLoS One..

[17] Hoa DT, Borjesson L, Nga NT, Johansson A, Malqvist M (2012). Sex of newborns associated with place and mode of delivery: a population-based study in northern Vietnam. Gend Med..

[18] Duong DM, Nguyen AD, Nguyen CC, Le VT, Hoang SN, Bui HTT (2017). A secular trend in birth weight and delivery practices in periurban Vietnam during 2005-2012. Asia Pac J Public Health.

[19] Goodkind DM (1995). Vietnam’s one-or-two-child policy in action. Population and Development Review..

[20] Nguyen HT, Eriksson B, Tran TK, Nguyen CT, Ascher H (2013). Birth weight and delivery practice in a Vietnamese rural district during 12 year of rapid economic development. BMC Pregnancy Childbirth..

[21] Toan TK, Eriksson B, An PN, NTK C, Bondjers G, Gottvall K (2013). Technology preference in choices of delivery care utilization from user perspective -a community study in Vietnam. Am J Public Health Res.

[22] Giang HTN, Ulrich S, Tran HT, Bechtold-Dalla PS (2018). Monitoring and interventions are needed to reduce the very high caesarean section rates in Vietnam. Acta Paediatr..

[23] World Population Review. Nha Trang Population 2020. https://worldpopulationreview.com/world-cities/nha-trang-population/ Accessed 27th Mar 2020.

[24] Matshidze KP, Richter LM, Ellison GT, Levin JB, McIntyre JA (1998). Caesarean section rates in South Africa: evidence of bias among different ‘population groups’. Ethnicity & health..

[25] Senaviratna NAMR, Cooray TMJ (2019). Diagnosing multicollinearity of logistic regression model. Asian Journal of Probability and Statistics.

[26] Le D-C, Kubo T, Fujino Y, Pham T-M, Matsuda S (2010). Health care system in Vietnam: current situation and challenges. Asian Pacific Journal of Disease Management..

[27] Rachatapantanakorn O, Tongkumchum P (2009). Demographic determinants for cesarean delivery in Pattani Hospital. Southeast Asian J Trop Med Public Health..

[28] Guilmoto CZ (2012). Son preference, sex selection, and kinship in Vietnam. Popul Dev Rev..

[29] Barbadoro P, Chiatti C, D’Errico MM, Di Stanislao F, Prospero E. Caesarean delivery in South Italy: women without choice: a cross sectional survey. PLoS ONE. 2012;7(9). 10.1371/journal.pone.0043906. 10.1371/journal.pone.0043906 PMC3444483 23028476

[30] Huang K, Tao F, Bogg L, Tang S. Impact of alternative reimbursement strategies in the new cooperative medical scheme on caesarean delivery rates: a mixed-method study in rural China. BMC Health Services Research. 2012;12(1). 10.1186/1472-6963-12-217. 10.1186/1472-6963-12-217 PMC3422992 22828033

